# Evaluating the effects of two different kinesiology taping techniques on shoulder range of motion and proprioception in patients with hypermobile Ehlers–Danlos syndrome: a randomized controlled trial

**DOI:** 10.3389/fresc.2024.1383551

**Published:** 2024-05-21

**Authors:** Frank Tudini, Max Jordon, David Levine, Michael Healy, Sarah Cathey, Kevin Chui

**Affiliations:** ^1^ Department of Physical Therapy, The University of Tennessee at Chattanooga, Chattanooga, TN, United States; ^2^ Healy Physical Therapy and Sports Medicine, East Providence, RI, United States; ^3^ Department of Physical Therapy, Radford University, Roanoke, VA, United States

**Keywords:** kinesiology tape, hypermobile Ehlers–Danlos syndrome, shoulder active range of motion, proprioception, physical therapy

## Abstract

**Background:**

Ehlers–Danlos syndrome (EDS) is a common group of inherited connective tissue disorders with a prevalence as high as 0.75%–2% of the population. Physical manifestations include pain and decreased proprioception, especially in more mobile joints, such as the shoulder. The kinesiology tape (K-Tape) is often used to treat patients with shoulder dysfunction. The effectiveness of the K-Tape is uncertain, and there is a lack of studies specifically studying the K-Tape in an EDS population.

**Purpose:**

The purpose of this study was to compare the short-term effects of two different K-Tape procedures on shoulder active joint reposition (AJR) and active range of motion (AROM) in patients with hypermobile EDS (hEDS) and shoulder pain.

**Methods:**

All participants were recruited from the EDS support groups and presented with shoulder pain. Baseline demographic information was obtained for each participant, after which AROM and AJR were assessed. The participants were randomized to receive one of two K-Tape procedures. Testing was repeated immediately post-taping and 48 h post-taping.

**Results:**

Significant improvements in shoulder external (*F* = 10.917, *p* < 0.001) and internal (*F* = 11.736, *p* < 0.001) rotations were seen from baseline to immediately post-taping and baseline to 48 h post-taping in the experimental K-Tape group. There were no significant differences in the shoulder rotation in the control K-Tape group and no significant differences in either group for shoulder flexion or AJR at any time point (*p* > 0.05).

**Conclusion:**

K-Tape may offer short-term improvements in shoulder rotation AROM in patients with hEDS and shoulder pain.

## Introduction

Ehlers–Danlos syndrome (EDS) is a common group of inherited connective tissue disorders estimated to affect as many as 0.75%–2% of the population (1). Hypermobile EDS (hEDS) is the most common subtype of EDS accounting for 80%–90% of all cases (2). hEDS is a clinical diagnosis based on several criteria, including joint hypermobility as assessed with a Beighton scale of ≥5 in adults, exclusion of other connective tissue disorders, and systemic manifestations, such as skin hyperextensibility and joint pain (3). Musculoskeletal pain affecting multiple joints, including the shoulder, is a common manifestation of hEDS, with some studies reporting joint pain in 100% of subjects (4, 5). Mobile joints, such as the shoulder, are particularly susceptible to pain in patients with hEDS, and this often occurs concomitantly with instability, decreased muscle strength, endurance, and proprioception (4–7), ultimately leading to decreased function. Additionally, even though shoulder instability is commonly found in hEDS, patients often present to physical therapy (PT) with a painfully limited and guarded active range of motion (AROM), further impacting function (1).

PT is a primary intervention for patients with hEDS and shoulder pain with treatment, including low-impact exercises for muscle strength and joint stabilization, proprioceptive training, and patient education (3, 8). The use of kinesiology tape (K-Tape) is a therapeutic technique often utilized to address shoulder dysfunction and alleviate pain. Theoretically, applying the elastic tape directly to the skin around or over the affected joint stimulates the afferent nerves and mechanoreceptors to decrease pain and enhance proprioception (9–11). The effectiveness of K-Tape for shoulder dysfunction is unclear, as is the mechanism through which it operates. With regard to proprioception, some studies have shown improvements in scapular joint position sense and movement control (12) and ankle inversion proprioception in patients with chronic ankle instability after K-Tape application (13). Other studies have shown that K-Tape improves neither proprioception in patients with chronic low back pain (14) nor knee proprioception in amateur runners (15). In addition, K-Tape has been shown to improve shoulder AROM in some studies (16) and did not provide increased benefit compared with exercise-based treatment in other studies (17). No studies examined the effects of K-Tape on proprioception and AROM in patients with hEDS. Therefore, the purpose of this study was to assess the short-term effects of two different K-Tape procedures on shoulder active joint reposition (AJR) and AROM in patients with hEDS and shoulder pain. It was hypothesized that K-Tape would improve both AJR and AROM.

## Materials and methods

The participants in this study were recruited from the EDS support groups in the New England area of the USA and were diagnosed with hEDS by their medical physician. The study was performed in a PT clinic in Rhode Island, USA, that specializes in treating individuals with hEDS. The inclusion criteria included a diagnosis of hEDS, unilateral or bilateral shoulder pain, a positive shoulder apprehension test, and a Beighton score of ≥5/9. The exclusion criteria included past shoulder surgery, cervical surgery, cervical injury within the last 12 months, and/or pregnancy. The study was approved by the University of Tennessee at Chattanooga Institutional Review Board (#20-040), and informed consent was obtained from all subjects.

Upon arrival and after gaining informed consent, the baseline demographic information for each participant was obtained. A computer-generated assignment to either the control or experimental K-Tape group was performed by an independent researcher. The participants were blinded to their allocation. Prior to taping, the shoulder AROM external rotation (ER), internal rotation (IR), elevation through flexion, and AJR were assessed in a supine position with a standard goniometer following a standardized procedure by the same examiner (18). The intra-rater reliability is good to excellent for measuring the shoulder IR, ER (19), and flexion in the supine position, with one study reporting ICC values between 0.94 and 0.99 (20). For AJR, the subjects' arms were placed in 90° of shoulder abduction, with the elbow bent to 90° of flexion and the forearm in pronation. The subjects were instructed to close their eyes. Their arms were then passively moved into 30° of ER and held stationary for 10 s while they were asked to concentrate on the position of their arms. The limb was then returned to the starting position and held for 5 s. They were asked to actively move their arms back to 30° of the ER position while keeping their eyes closed. The number of degrees away from 30° of ER was documented as an AJR error (21). Following these measurements, K-Tape was applied to the painful shoulder(s). AROM ER, IR, and elevation through flexion, as well as AJR, were re-evaluated immediately post-taping and again 48 h post-taping. K-Tape was then removed, and the skin was assessed for any adverse reactions.

**Figure 1:**
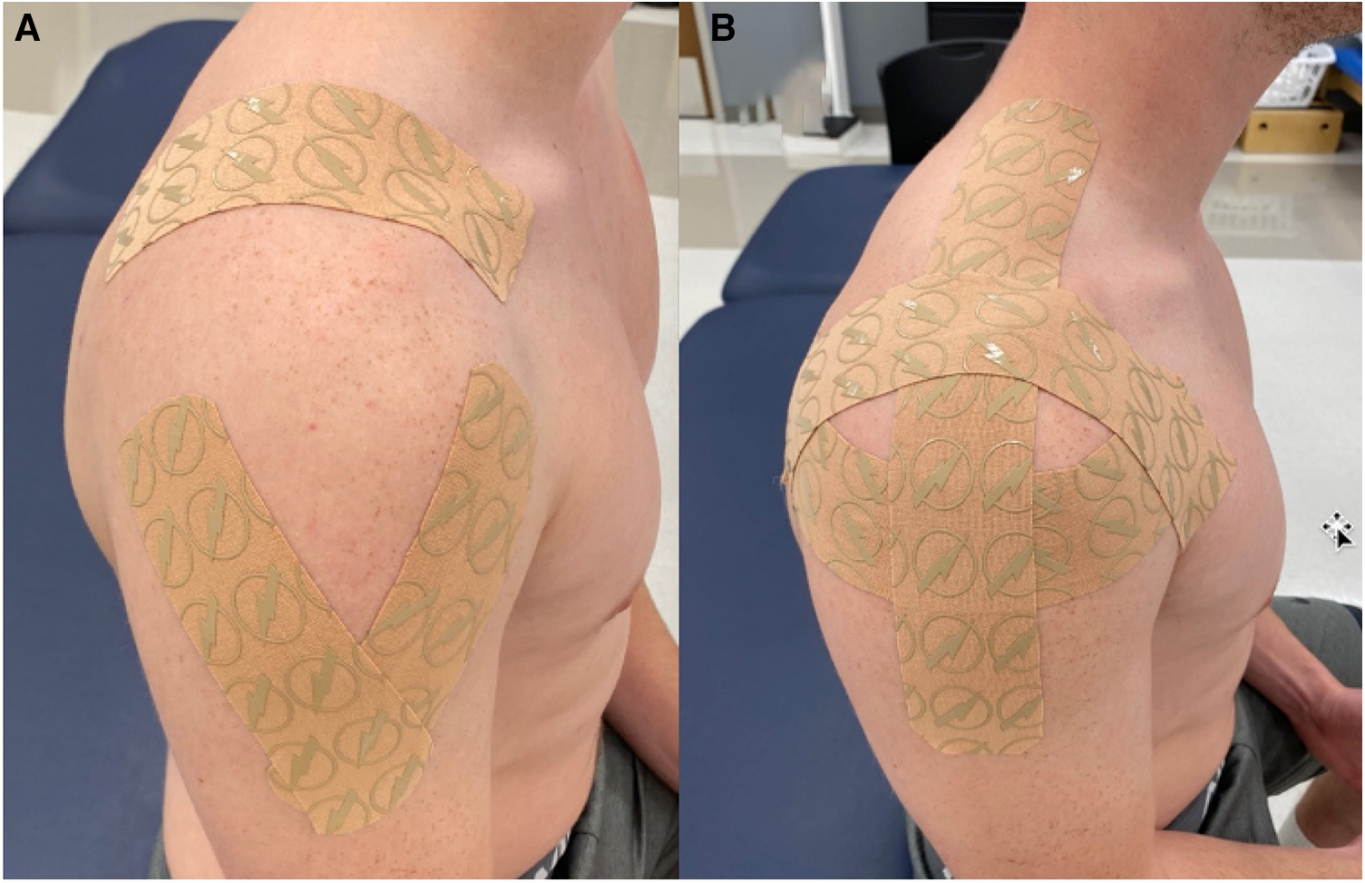
(**A**) Control K-Tape group. (**B**) Experimental K-Tape group.

All subjects presented with bilateral shoulder pain; therefore, both shoulders were taped using the same taping protocol by the same therapist. All taping utilized the Thrive Far Infrared Kinesiology Tape (Melrose, MA, USA). The taping techniques have been previously described in the literature (22). However, in brief, the experimental group had the first strip applied from the lateral inferior aspect of the clavicle wrapping around to the medial posterior scapular. The second strip was applied proximal to distal over the upper trapezius muscle ending around the deltoid tuberosity. The third strip ran from the anterior deltoid over the upper trapezius ending near the spine of the scapula. The first strip of the control K-Tape group was applied from the anterior deltoid to the deltoid tuberosity, followed by a strip from the posterior deltoid to the deltoid tuberosity forming a “V” over the lateral arm. The third strip ran over the upper trapezius from the clavicle to the spine of the clavicle. Both groups received three strips of tape, with the main difference between the protocols being that the control group’s tape did not cross the GH joint line (Figure 1).

## Statistical approach

Data were analyzed using SPSS (IBM SPSS Statistics for Windows, Version 26.0). Demographic data (i.e., age, height, weight, and gender) were analyzed using a series of independent samples *t*-tests and Fisher's exact test to determine if the groups were similar at baseline. To analyze the effects of K-Tape on shoulder AROM and AJR, a 3 × 2 × 2 (time × tape × shoulder) mixed ANOVA was performed for each dependent variable. For significant results, a *post hoc* analysis using the least squared difference was used to determine where the differences were. An alpha level of 0.05 was used to determine statistical significance.

## Results

**Figure 2:**
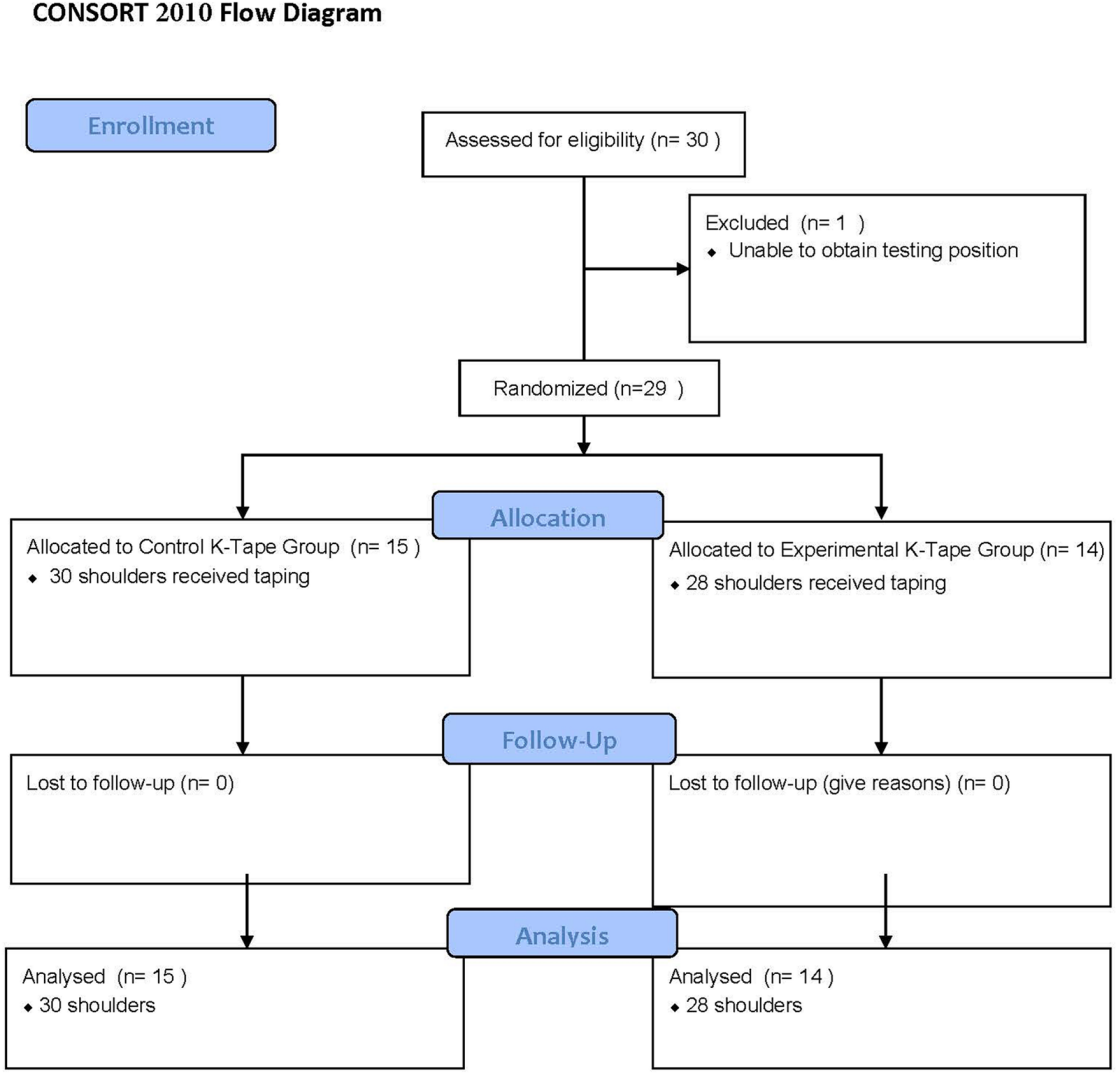
Consort flow diagram.

A total of 30 subjects were recruited. However, one subject was unable to participate due to failure to achieve 90° of shoulder abduction, a prerequisite for the shoulder rotation AROM and AJR measurements. A total of 29 participants, representing 58 shoulders as all subjects had bilateral shoulder pain, were included (15 in the control K-Tape group and 14 in the experimental K-Tape group) (Figure 2) (23). Demographic information is presented in Table 1. The groups were normally distributed for parametric analysis, as assessed by the Shapiro–Wilk test for normality. The independent samples *t*-test demonstrated that there were no significant differences between groups at baseline for age, height, and weight. Fisher's exact test was used for gender; however, only 1 of the 29 participants was male (Table 1). This was not unexpected as the majority of people diagnosed with hEDS are female (24). All subjects wore the tape through the 48 h re-assessment. Skin fragility is a common finding in patients with EDS (25), and in 5/58 (5.2%) shoulders, minor adverse skin reactions occurred. These subjects were contacted via phone 4 days post-tape removal, and all adverse skin reactions had resolved.

**Table 1:** Demographic information.

Demographic	*n*	Group	Measure	*t*-test or Fisher's exact test (*p*-value)
Age (years)	14	Experimental	35.23 (10.76)	−2.04 (0.051)
	15	Control	47.33 (17.97)	
Gender	14	Experimental	1 male, 13 female	(0.48)
	15	Control	0 male, 15 female	
Height (cm)	14	Experimental	159.72 (8.10)	−2.01 (0.055)
	15	Control	165.18 (5.64)	
Weight (kg)	14	Experimental	62.45 (14.37)	−1.76 (0.09)
	15	Control	74.57 (8.15)	

*n* = number of subjects.

The AROM ER, IR, and flexion values taken at baseline, immediately post-taping, and 48 h post-taping are presented in Table 2. The 3 × 2 × 2 (time × tape × shoulder—side left vs. right) mixed ANOVA comparing AROM and AJR across time (i.e., pre-taping, immediately post-taping, and 48 h post-taping), techniques (experimental vs. control), and shoulders (right vs. left) is presented in Table 3. The mixed ANOVA analyzing ER AROM showed a significant main effect for time (*F*_2,104_ = 20.533, *p* < 0.001, *η*^2^_ _=_ _.283) and the interaction term time × tape (*F*_2, 104_ = 10.917, *p* < 0.001, *η*^2^_ _= .174). The ER AROM trended upward over all three time points in both groups. However, *post hoc* analysis revealed significant increases only from baseline to immediately post-taping and baseline to 48 h post-taping for the experimental K-Tape group. The mixed ANOVA analyzing IR AROM showed a significant main effect for time [*F*_(2, 104)_ = 9.094, *p* < 0.001, *η*^2^_ _= .149] and the interaction term time × tape [*F*_(2, 104)_ = 11.736, *p* < 0.001, *η*^2^_ _= .184]. The *post hoc* analysis revealed a significant improvement in IR AROM in the experimental K-Tape group from baseline to immediately post-taping and from baseline to 48 h post-taping. There was no significant improvement at any time point in the control K-Tape group. The mixed ANOVA analyzing flexion AROM showed no significant differences in AROM at any time point or between the K-Tape groups. In addition, there were no significant differences in AJR sense at any time point for either group (Table 4).

**Table 2:** Range of motion in degrees.

Measure	Group	*n*	Shoulder	Baseline mean (SD)	Immediate post-taping mean (SD)	48 h post-taping mean (SD)
AROM ER	Experimental	4	Right	68.08 (20.26)	77.62 (17.75)	79.23 (17.95)
			Left	62.85 (24.34)	71.15 (20.64)	75.77 (16.41)
	Control	15	Right	66.47 (20.21)	66.93 (19.73)	68.67 (18.90)
			Left	62.00 (21.29)	63.60 (21.83)	63.87 (20.74)
AROM IR	Experimental	14	Right	69.00 (18.70)	77.62 (14.33)	80.00 (8.90)
			Left	69.15 (11.78)	74.77 (11.37)	76.08 (10.74)
	Control	15	Right	64.53 (15.47)	60.87 (19.10)	66.07 (15.85)
			Left	65.33 (17.20)	62.20 (19.43)	65.20 (16.28)
AROM Elevation	Experimental	14	Right	159.00 (18.22)	165.54 (17.87)	166.23 (19.61)
			Left	147.15 (35.03)	156.23 (32.09)	160.31 (28.85)
	Control	15	Right	142.87 (38.60)	146.73 (37.25)	139.47 (38.64)
			Left	139.20 (38.45)	139.73 (39.74)	140.80 (37.21)

AROM, active range of motion; *n*, number of subjects; SD, standard deviation.

**Table 3:** 3 × 2 × 2 (time × tape × shoulder) mixed ANOVA comparing the active range of motion and active joint repositioning test across time (pre-taping, immediately post-taping, and 48 h post-taping), techniques (experimental vs. control), and shoulders (right vs. left).

Variable	Time (*F*-value) (*p*-value)	Time × technique (*F*-value) (*p*-value)	Time × shoulder (*F*-value) (*p*-value)	Time × tape × shoulder (*F*-value) (*p*-value)
AROM ER	**20.553** **(<0.001)**	**10.917** **(<0.001)**	0.72 (0.93)	0.490 (0.61)
AROM IR	**9.094** **(<0.001)**	**11.736** **(<0.001)**	0.795 (0.45)	.312 (0.73)
AROM elevation	2.083 (0.13)	2.055 (0.13)	0.716 (0.49)	0.167 (0.85)
AJR ER	0.141 (0.87)	1.669 (0.19)	0.574 (0.57)	1.783 (0.17)

AROM, active range of motion; ER, external rotation; IR, internal rotation; AJR, active joint reposition.

The bold values represent statistical significance.

**Table 4:** Active joint reposition error in degrees.

Measure	Group	*n*	Shoulder	Baseline mean (SD)	Immediately post-taping mean (SD)	48 h post-taping mean (SD)
AJR	Experimental	14	Right	5.31 (4.25)	6.23 (4.68)	5.23 (3.77)
			Left	7.46 (3.28)	5.54 (3.73)	3.69 (3.40)
AJR	Control	15	Right	6.40 (3.11)	6.33 (4.12)	5.80 (5.77)
			Left	6.27 (4.57)	5.87 (4.26)	12.27 (21.95)

AJR, active joint reposition, SD, standard deviation.

A *post hoc* power analysis was performed for the interaction of time by tape for each outcome of interest to assess the likelihood of a Type II error. For each power analysis, the effect size was taken from an unreported 3 × 2 mixed ANOVA, with the right and left shoulder variables collapsed due to the absence of significant differences between them. Furthermore, a total sample size of 58 was used (total number of shoulders), with three measurements and an assumption of a correlation among repeated measures of 0.5 and a non-sphericity correction of 1. The power analysis demonstrated adequate power for ER AROM (1−β = 1), IR AROM (1−β = 1), flexion AROM (1−β = 0.908), and AJR ER (1−β = 0.834). Therefore, we feel confident that a Type II error was not committed.

## Discussion

Although all subjects were hypermobile, shoulder AROM was less than would be expected and less than the standard measurements expected for a healthy population. Subject AROM may have been limited by pain, apprehension, fear of joint instability, muscle guarding, or a combination of these factors. The primary purpose of this study was to assess the effects of two different K-Tape procedures on AROM and AJR in patients with hEDS and shoulder pain. While both K-Tape groups showed minor improvements in ER over all three time points, significant improvements were seen only in the experimental K-Tape group from baseline to immediately post-taping and from baseline to 48 h post-taping. The improvement from baseline to each time point in the experimental K-Tape group surpassed the measurement error of 7.48° (19). This is important as normal ER ROM is cited as 90° (18), and this could be conceivably higher in a hypermobile population. In the experimental K-Tape group, the subjects averaged only 65.47° of ER at baseline (Table 2). While far less than normal values, the amount of ER range needed for some common activities of daily living (ADLs) has been documented as 59° ± 10 (26). This is meaningful as the ER testing occurred at 90° of shoulder abduction, a common position for apprehension, dislocation, and subluxation (27). Prior to testing, each subject had a positive apprehension test. The improvement in the experimental group to an average of 74.4° of ER immediately post-taping and 77.5° 48 h post-taping indicated that the subjects felt more comfortable and stable moving into this range. Interestingly, ER AROM, which was the most limited at baseline, displayed the greatest improvement of the three measured motions.

Normal IR AROM is cited as 70°–90° (18). At baseline, the subjects in the experimental K-Tape group averaged 69.1° of IR. There was a significant improvement in IR AROM in this group from baseline to immediately post-taping (76.2°) and from baseline to 48 h post-taping (78.04°), surpassing the minimal detectable change of 4.02° (19) (Table 2). The control K-Tape group experienced a decrease in IR AROM from an average of 64.9° at baseline to an average of 61.5° immediately post-taping, with a return to baseline values with an average of 65.6° of IR 48 h post-taping.

Normal shoulder flexion in adults is traditionally cited as varying between 165° and 180° (18). However, more recent research has shown that, in the general population of 30–40-year-old adults, flexion AROM is closer to 158°–168° (28). At baseline, our subjects averaged 147° of shoulder flexion. The experimental K-Tape group demonstrated minor improvements at each time point, from an average of 153° at baseline to 160° immediately post-taping and 163.3° 48 h post-taping. Although these were not statistically significant, the measurements from baseline to immediately post-taping and from baseline to 48 h post-taping surpassed the standard error of measurement of 2.7° (29), and the ranges were comparable to the general population averages stated above. Conversely, the control K-Tape group averaged 141° at baseline and improved to 143.23° immediately post-taping but then decreased to 140.1° 48 h post-taping, placing them well below general population averages. It should be noted that only approximately 130° of shoulder elevation is needed for most functional ADLs, with a maximum of 142° required to reach toward high shelves (30). Thus, the patients in both groups demonstrated functional or close to functional AROM flexion for most ADLs even prior to taping (Table 2).

Decreased proprioception has been found in patients with hEDS (31) and has been associated with activity limitations (7). Proprioceptive deficits have also been documented in patients with shoulder pain (32) and in those with shoulder instability (33, 34), hallmarks of hEDS, and features present in all subjects within this study. Assessing AJR sense is a common method of assessing proprioception. The procedure utilized is well established in the literature (21, 35); however, a standard goniometer was used in place of an isokinetic dynamometer. This change was purposeful, such that testing could be readily performed in any clinic. The small error of measurement and the high intra-rater reliability of shoulder goniometry helped to ensure accurate results (19). At baseline, the average AJR error for all subjects was 6.36°. The normal values for AJR ER at 30° of ER were found in one study to range from 3.1 to 3.6 ± 0.9 to 1.5 depending on gender and dominance using a high-accuracy computer-controlled electronic goniometer (34). Another study reported an AJR of 1.13 ± 0.32° at 20° of ER using a digital goniometer (36). Most of the articles we found demonstrating a proprioceptive deficit in people with hEDS were performed on the lower extremity, primarily the knee (7, 37, 38). Our results indicate that a proprioceptive deficit in people with hEDS is also present in the shoulder. However, K-Tape did not improve AJR in this study: There were no significant differences in AJR at any time point in either group. These results are contrary to those of some studies that assessed the effects of K-Tape on shoulder proprioception in healthy subjects (11) but align with other studies and systematic reviews that showed no improvement in proprioception with K-Tape in patients with shoulder pain (39) and other proprioceptive deficits (40).

It must be acknowledged that proprioception is a global term encompassing more than AJR (41). Movement, vibration, force, passive joint positioning, and muscular effort are all perceived through sensory and mechanical receptors located in soft tissue (41). While AJR did not improve with K-Tape, other domains of proprioception may have benefitted but were untested. AJR was chosen for this study due to its relationship with function and the sensitivity it demonstrates due to the concurrent stimulation of capsuloligamentous and musculotendinous mechanoreceptors (42). It is possible that, due to joint laxity, proprioceptors were not stretched in our AJR testing position, which could have led to the findings. Nonetheless, based on these findings, we cannot recommend K-Tape to improve shoulder AJR in people with hEDS.

While our study cannot answer the mechanism through which the K-Tape application improved AROM rotation, we can state that it does not appear to be through improving AJR. In a previous study, using the same taping protocols in patients with hEDS and shoulder pain, it was demonstrated that there was a significant short-term improvement in pain and function after either K-Tape application with no difference between the groups (22). However, the results of this study favor the experimental tape, indicating that pain reduction may not be the only mechanism for improving the rotational AROM in the experimental K-Tape group. The primary difference between the two taping protocols was that the experimental tape crossed the shoulder joint, whereas the control tape did not. It is unlikely that any direct mechanical benefit to the joint would result from elastic tape applied with little tension; hence, there may be a neuromuscular effect or increased sensory input around the joint, which may stimulate cutaneous and muscle mechanoreceptors by stretching the skin during motion (43). The experimental tape encompassed the shoulder joint more completely, and, theoretically, this may have caused more biomechanical skin deformation, particularly during internal and ER motions (44). Furthermore, studies examining the ankle plantar flexors have shown that K-Tape may affect muscle activity, as seen in one study analyzing the EMG activity during a vertical jump (45) and in other studies showing an effect on the H-reflex (46, 47). However, more research is needed in this area. We also cannot rule out psychological effects, placebo, and the Hawthorne effect (48); however, if these were present, they would likely have equally affected both K-Tape groups.

Our results, combined with the low risk of adverse side effects, suggest that K-Tape may be a viable short-term treatment option for those with hEDS with pain and limited shoulder rotation motion. We would further recommend that the tape cross the glenohumeral joint. To further decrease the risk of adverse skin reactions, the K-Tape should be applied with minimal stretch, although there may be a difference in response based on the brand of tape used.

Our study has limitations. Both groups received a taping protocol (either control or experimental); thus, we did not have a true control group. It is possible, although unlikely, that the subjects may have researched different taping techniques for the shoulder to self-determine if they were in the control or experimental group, which may have affected their response to the treatment. Additionally, there may have been slight variations in the tape application between shoulders and participants; however, this was minimized by having the same researcher, who was experienced with K-Tape, perform all taping procedures. There was a small sample size of 29 participants, although with all subjects having bilateral shoulder pain, this represented 58 shoulders. The testing period was limited to 48 h, so we are unable to speculate on any longer-term effects of K-Tape. While the procedure used to assess AJR is established in the literature, we did not use an isokinetic or digital dynamometer, opting for a standard goniometer to reflect the resources in a typical clinical setting. While the accuracy and the intra-rater reliability of the goniometric assessment of the shoulder are well established, this method of measuring AJR has not been validated, and our results may not be as accurate as those obtained using other methods for measuring AJR.

## Conclusion

hEDS is a common condition manifesting with decreased proprioception and shoulder AROM. K-Tape crossing the glenohumeral joint is an inexpensive and relatively safe intervention that may offer temporary improvements in shoulder rotation AROM. The improvement in AROM does not appear to be related to any effect on AJR.

## Data Availability

The original contributions presented in the study are included in the article/Supplementary Material. Further inquiries can be directed to the corresponding author.
